# Understanding the Significance of Layer Bonding in Melt Electrowriting

**DOI:** 10.1002/advs.202407514

**Published:** 2024-10-24

**Authors:** Christopher D. Lamb, Brooke Maitland, Matt S. Hepburn, Tim R. Dargaville, Brendan F. Kennedy, Paul D. Dalton, Adrian Keating, Elena M. De‐Juan‐Pardo

**Affiliations:** ^1^ T3mPLATE Harry Perkins Institute of Medical Research QEII Medical Centre Nedlands and Centre for Medical Research The University of Western Australia Perth WA 6009 Australia; ^2^ School of Engineering The University of Western Australia Perth WA 6009 Australia; ^3^ BRITElab Harry Perkins Institute of Medical Research QEII Medical Centre Nedlands and Centre for Medical Research The University of Western Australia Perth WA 6009 Australia; ^4^ Institute of Physics, Faculty of Physics, Astronomy and Informatics Nicolaus Copernicus University in Torun Grudziadzka 5 Torun 87‐100 Poland; ^5^ Centre for Materials Science School of Chemistry and Physics Faculty of Science Queensland University of Technology Brisbane QLD 4000 Australia; ^6^ Phil and Penny Knight Campus for Accelerating Scientific Impact University of Oregon Eugene OR 97403 USA; ^7^ Curtin Medical School Curtin University Perth WA 6102 Australia

**Keywords:** 3D printing, layer bonding, mechanical properties, melt electrowriting, tissue engineering

## Abstract

Melt electrowriting (MEW) is a high‐resolution additive manufacturing technology capable of depositing micrometric fibers onto a moving collector to form 3D scaffolds of controlled mechanical properties. While the critical role of layer bonding to achieve mechanical integrity in fused deposition modeling has been widely reported, it remains largely unknown in MEW, in part due to a lack of methods to assess it. Here, a systematic framework is developed to unravel the significance of layer bonding in MEW scaffolds and its ultimate effect on their mechanical properties. Results show that printing parameters, scaffold design, and print path have a strong impact on layer bonding strength of poly(ɛ‐caprolactone) MEW scaffolds. This study demonstrates that a small increase of 5 µm in fiber diameter can enhance the layer bonding strength by as much as 70%, greatly impacting the overall scaffold properties. A method is also established to control MEW scaffold layer bonding using a heated collector. Importantly, this study reveals that scaffold architecture alone is not responsible for the overall mechanical properties. Finally, a method to obtain tailored layer bond strengths within a given scaffold is established. This has significant implications as provides new possibilities to control mechanical properties of MEW scaffolds through layer bonding.

## Graphical abstract

Melt electrowriting (MEW) is an advanced additive manufacturing technique that prints micrometric fibers to create tailored porous scaffolds. This study develops a framework to investigate the bonding strength between layers, finding that factors like fiber diameter and collector temperature have a profound effect on overall scaffold performance.

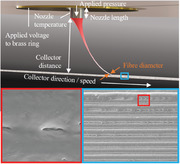

## 1 Introduction

Melt electrowriting (MEW) is a high‐resolution additive manufacturing technique capable of depositing polymeric fibers onto a moving collector with high accuracy.^[^ ^1^, ^2^ ^]^ One of the main advantages of MEW scaffolds is that they can be tailored to mimic the complex biomechanical response of tissues^[^ ^3^, ^4^, ^5^ ^]^ and guide cellular responses in tissue engineering applications.^[^ ^6^, ^7^, ^8^ ^]^ MEW integrates principles of fused filament fabrication (FFF) and electrospinning to print micro to nano‐scale fiber sizes (100 µm–350 nm).^[^ ^9^, ^10^ ^]^ To achieve such small fiber, an electric field is created by the application of a high voltage to the nozzle and a grounded collector to stretch the molten polymer jet.^[^ ^11^, ^12^ ^]^ Extrusion is achieved at minute flow rates typically using pneumatics applied to a syringe,^[^ ^2^, ^13^ ^]^ but can also be performed by direct drive mechanics.^[^ ^14^ ^]^

To obtain high‐quality prints, it is necessary to carefully adjust the processing conditions to ensure a constant, stable jet. There are multiple processing parameters that impact the quality of the fabricated MEW scaffold. The primary parameters are nozzle diameter, applied voltage, nozzle temperature, pressure, collector speed, and collector distance.^[^ ^11^, ^15^, ^16^, ^17^ ^]^ Collector distance refers to the gap between the print head and the collector, as unlike other extrusion‐based 3D printing methods such as FFF, MEW is a non‐contact printing process.^[^ ^15^, ^18^ ^]^ The fiber diameter is controlled by all primary processing parameters, with the most influential being applied pressure and nozzle diameter.^[^ ^19^, ^20^, ^21^ ^]^ As the polymer melt leaves the nozzle, the electrostatically charged jet gets stretched under the effect of the electric field and thus diameter reduction is the greatest directly beneath the print head. Once the jet is deposited onto the collector and the material has cooled, further fiber diameter changes are minimal.^[^ ^18^, ^19^ ^]^ Understanding the independent effect of each print parameter on the printed scaffold is difficult due to the interrelated nature of MEW.^[^ ^11^, ^19^ ^]^ It is known that small changes in pressure or nozzle temperature significantly impact the scaffold fiber diameter, whilst other printing parameters such as applied voltage, collector speed, and collector distance also do though to a much‐reduced degree.^[^ ^11^, ^22^, ^23^ ^]^

In MEW printing, the polymer jet is deposited a short distance (lag distance) behind the nozzle.^[^ ^2^, ^11^, ^19^, ^24^ ^]^ Minimizing this lag distance is often preferable to ensure fidelity of the desired print path.^[^ ^25^, ^26^ ^]^ Scaffolds produced by MEW are usually fabricated using a single repeated pattern over multiple layers, where the pattern is defined by the 2D movement of the collector with the third dimension provided by the vertical stacking of layers.^[^ ^15^, ^25^, ^26^, ^27^ ^]^ This results in the doubling of layers where the fiber path crosses due to the larger collector distance, which is unlike FFF where each layer of the printed material is physically flattened by the nozzle itself.^[^ ^17^, ^27^ ^]^ Furthermore, the fiber spacing resolution of these patterns is affected by both fiber diameter and applied voltage, as the landing jet is subjected to repulsion forces by the residual charge accumulated in previously deposited layers, which can lead to defects such as fiber bridging.^[^ ^12^, ^18^, ^28^ ^]^ These residual charge effects make the manufacturing of thick, high‐layered scaffolds increasingly challenging.^[^ ^17^, ^29^ ^]^

The design of the MEW scaffold has a crucial impact on the final mechanical properties, particularly scaffold pattern, fiber spacing (often called pore size), and the number of layers.^[^ ^17^, ^30^, ^31^, ^32^ ^]^ Layer bonding refers to the process of joining or fusing multiple layers and is an inherent phenomenon of both FFF and MEW, as well as other types of 3D printing and material synthesis.^[^ ^33^, ^34^ ^]^ Layer bonding in FFF has been extensively investigated^[^ ^33^, ^35^ ^]^ with the help of non‐destructive imaging techniques such as optical coherence tomography (OCT),^[^ ^36^, ^37^ ^]^ to ensure the layers had correctly bonded to produce a solid uniform part.^[^ ^38^, ^39^, ^40^ ^]^ Increases in FFF layer bonding have been achieved by increasing either collector and/or nozzle temperature to enhance mechanical integrity.^[^ ^3^, ^34^, ^41^, ^42^, ^43^, ^44^, ^45^ ^]^ Recently, OCT has also been proposed as a potential tool to assess layer bonding in MEW scaffolds.^[^ ^46^ ^]^ However, the importance of layer bonding in MEW scaffolds remains largely unknown.^[^ ^3^, ^23^, ^41^, ^42^ ^]^ This study aims to examine the significance of layer bonding and its ultimate effect on scaffold mechanical properties in MEW printing of poly(ɛ‐caprolactone) (PCL) and identify methods to control it.

## 2 Results and Discussion

### 2.1 Effect of Primary Processing Parameters on Layer Bonding

**Figure 1:**
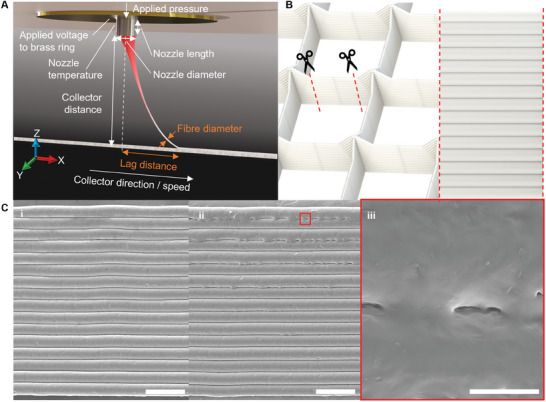
Proposed framework to assess layer bonding in melt electrowriting (MEW). A) Rendered schematic of MEW jet and fibers, showing all primary processing parameters that can be controlled by the user. B) Render of MEW scaffold with square pores with 1 mm spacing, dashed lines show location of cuts to remove wall for imaging with a render of the cut section of the scaffold wall. C) Scanning electron microscopy (SEM) image of 16 stacked fibers forming a scaffold wall. i) Stacked fibers showing no bonding across fibers. Scale Bar = 100 µm. ii) Stacked fibers showing moderate layer bonding at the top of the scaffold wall. Scale Bar = 100 µm. iii) Enlarged section of white rectangle shown in ii, showing magnified view of layer bonding. Scale bar = 20 µm.

With MEW printing it is important to understand the influence of each printing parameter on the final properties of the printed constructs. However, it is not feasible to assess the impact of each single printing parameter individually, as they have synergistic effects.^[^ ^47^ ^]^ In order to investigate the impact of MEW primary processing parameters on layer bonding, schematically shown in **Figure** **1**A, determination of stable printing conditions is essential to achieve high print quality and minimal printing defects.^[^ ^21^ ^]^ This requires extensive empirical work, as well as continual monitoring of the printing conditions to avoid pulsing.^[^ ^20^ ^]^ Thus, we first developed a computational framework combined with computer vision to assess the stability of the jet and the approximate fiber diameter using high‐throughput methodology. We used the framework to identify stable printing conditions and investigate layer bonding in scaffolds with two different fiber diameters (25 and 31 µm) known for good cell growth^[^ ^9^ ^]^ by adjusting the collector speed and distance while keeping, nozzle temperature, nozzle diameter, applied pressure and voltage constant, as changing them would also change fiber diameter. This approach allowed for a systematic evaluation of the relationship between the selected processing parameters and the resulting layer bond strength. A summary of the investigated parameter values is presented in **Table** **1**.

**Table 1:** The experimental printing parameters used for each of the different scaffolds tested.

Fiber diameter (µm)	25 ± 1	25 ± 1	31 ± 1	31 ± 1
Collector speed (mm/min)	300	600	300	600
Collector distance (mm)	3–4	3–4	3–4	3–4
Lag distance (mm)	1.2	1.2	1.2	1.2
Collector temperature (°C)	26	26	26	26

In order to assess the layers in a fiber wall of a MEW scaffold, a cross‐section of the scaffold wall was removed and mounted flat for scanning electron microscopy (SEM) analysis, as previously shown^[^ ^48^ ^]^ and illustrated in Figure 1B. An example of a resulting cross‐section is shown in Figure 1C. SEM images revealed scaffold walls with i) almost no layer bonding, compared to ii, iii) showing more bonding at the top of the wall, demonstrating a bottom‐to‐top increased gradient of bonding across the scaffold wall.

**Figure 2:**
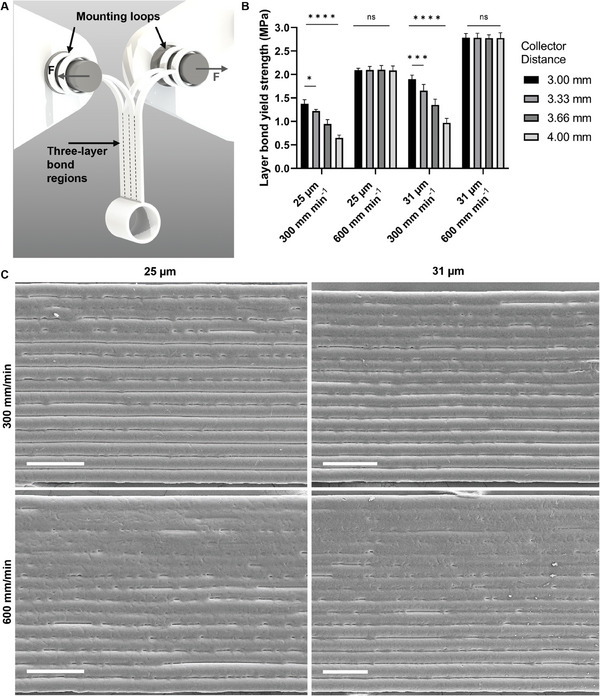
Uni‐axial layer bond yield strength mechanical testing of MEW scaffolds and SEM images. A) Render of designed scaffolds to investigate their layer bond strength via uniaxial tensile testing. B) Layer bond yield strength as a function of fiber diameter, collector speed, and collector. All data shows average over *n* = 5, error bars represent one standard deviation, and significant differences were assessed using two‐way ANOVA with Tukey's multiple comparisons test. Statistical significance is shown at ^*^ *p* ≤ 0.05, ^**^ *p* ≤ 0.01, ^***^ *p* ≤ 0.001, ^****^ *p* ≤ 0.0001, ns = not significant C) SEM images comparing scaffolds printed at two different collector speeds (300 and 600 mm min^−1^) and with two fiber diameters (25 and 31 µm); collector distance was kept constant at 3.00 mm. All scale bars = 100 µm.

To quantitatively determine the layer bond yield strength, a custom scaffold was adapted from *Bakirci*, et al. to allow shearing between layers when uniaxially tensioned (**Figure** **2**A).^[^ ^44^ ^]^ Each scaffold was mounted onto a uniaxial tensile tester using mounting loops.^[^ ^49^ ^]^ The scaffolds were printed in a way so that groups of 4 fibers could be attached to alternating mounting loops. This allowed for the bonding between three layers to be tested in a single test, thereby improving the signal‐to‐noise in the stress‐strain curves. Through isolating and controlling the key parameters, this study aimed to accurately assess the impact of layer bonding in the process. This approach allowed for a systematic evaluation of the relationship between the selected processing parameters and the resulting layer bond strength.

Four collector distances (3.00, 3.33, 3.66, and 4.00 mm), were investigated in the study. As the collector distance increased, there were significant decreases in layer bond yield strength at slower print speeds (300 mm min^−1^) (Figure 2B). This effect was consistent for both fiber diameters, with each diameter experiencing a significant 70% drop in strength when the collector distance increased from 3 to 4 mm. This decrease in layer bond yield strength with increased collector distance, is due to the additional time the fiber has to cool down prior to contact with the previous layer.

The enhanced layer bond yield strength of the 31 µm fibers could be attributed to the increased volume‐to‐surface area ratio. Comparing the 25 to 31 µm diameters, the surface area increases by 17%, while the cross‐sectional area expands by 31%. This greater cross‐sectional area could be responsible for the improvement in strength, likely due to the increased heat capacity.

Interestingly, the percentage increase in layer bond strength from 25 to 31 µm fiber diameter is ≈72%, which closely aligns with the percentage change in cross‐sectional area. This could suggest a relationship between the layer bond yield strength and cross‐sectional area for these specific fiber diameters. Exploring the relationship between layer bond yield strength and cross‐sectional area in future would be useful to determine if a similar relationship to the volume‐to‐surface area ratio is observed (Figure S1, Supporting Information).

Increasing the collector speed from 300 to 600 mm min^−1^, resulted in a significant percentage change in layer bond yield strength of 150% to 300%. This change can be primarily attributed to the reduced time between the deposition of consecutive layers, which in turn reduces the cooling time available for the deposited material.

Notably, for both fiber diameters at a collector speed of 600 mm min^−1^, there was no change with variations in collector distance. From the mechanical testing videos, it was revealed that the fibers were failing in tension before the layer bonds (Figure S2, Supporting Information). This failure occurred due to necking of the fibers,^[^ ^50^ ^]^ which occurred prior to de‐bonding of the fibers.

Analysis of the SEM images at the 3 mm collector distance for both fiber diameters and collector speeds visually demonstrates the increase in layer bonding (Figure 2C). The increased layer bonding is shown in Figure 2C‐iii,iv (printed at 600 mm min^−1^), by the reduced distinction of layer lines compared with Figure 2C‐i,ii. All the SEM images highlight the influence of heat from the print head via gradient of fusion shown in the wall, where the bottom of each image corresponds to the base of the scaffold wall. This gradient is due to the fibers that are deposited onto the top of the wall having a reduced time to cool and therefore undergo increased bonding to the already deposited fibers.

### 2.2 Effect of Print Path and Collector Temperature on Layer Bonding

We then examined the influence of the print path and collector temperature on layer bond strength. In order to do so, we designed a set of experiments with the same scaffold design previously used, where we introduce a deposition delay. To ensure optimal sensitivity of measurement, we selected the parameter combination that generated the highest bond layer yield strength based on the previous findings (collector distance = 3.00 mm, collector speed = 300 mm min^−1^, fiber diameter = 31 µm, and lag distance = 1.2 mm).

Collector temperature is an independent processing parameter that does not influence any of the primary parameters and has been previously shown to improve deposition accuracy.^[^ ^16^ ^]^ For this analysis, we chose five collector temperatures, ranging from 26 to 46 °C with increments of 5 °C. This range was chosen to ensure that the sample temperature stayed below the melt temperature of PCL, accounting for the extra heat irradiated from the print head.^[^ ^51^ ^]^

**Figure 3:**
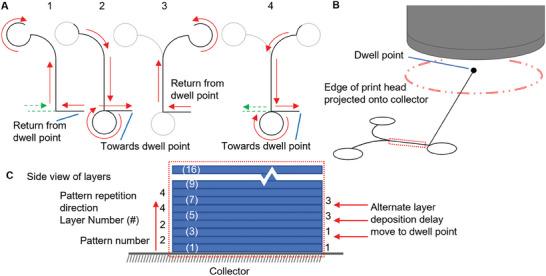
Diagram of printing path and print pattern for the scaffolds prepared to assess layer bond strength. A) Diagram of G‐code print path for the mechanical testing scaffold, steps 1–4 are completed as a loop to produce 1 layer pair. The dashed line shows the entry and exit point for the scaffold between steps 2 and 3 where the print head is moved away from the scaffold. B) Diagram showing the location of the dwell point to move the scaffold outside the print head area. C) Side view of the layers to show how the first 8 layers are printed.

When producing large scaffolds, there is often a long delay between the deposition of consecutive layers. During these intervals, certain points in the scaffold remain exposed to the atmospheric conditions from outside of the effective print head area. To simulate this scenario, we incorporated a dwell point between every second layer, considering the repetitive pattern of the scaffold (**Figure** **3**A). The dwell point was positioned at a distance that ensured that the entire scaffold was outside the print head region, thereby exposing it to the surrounding air (Figure 3B). The duration of time spent at the dwell point varied from 0 to 100 s in increments of 25 s. In cases where the time away from the print path was 0 s, no alteration was made to include a dwell point.

To establish three distinct bond regions for analysis, we implemented an optimized print order strategy. This involved a repetitive pattern of two left loops followed by two right loops, resulting in a total of eight loops spanning across 16 layers (Figure 3C). The starting and ending points of the print head on the scaffold are indicated by the dashed line. For a more comprehensive understanding of the printing process, an animation demonstrating the entire procedure is provided in (Video S1, Supporting Information).

**Figure 4:**
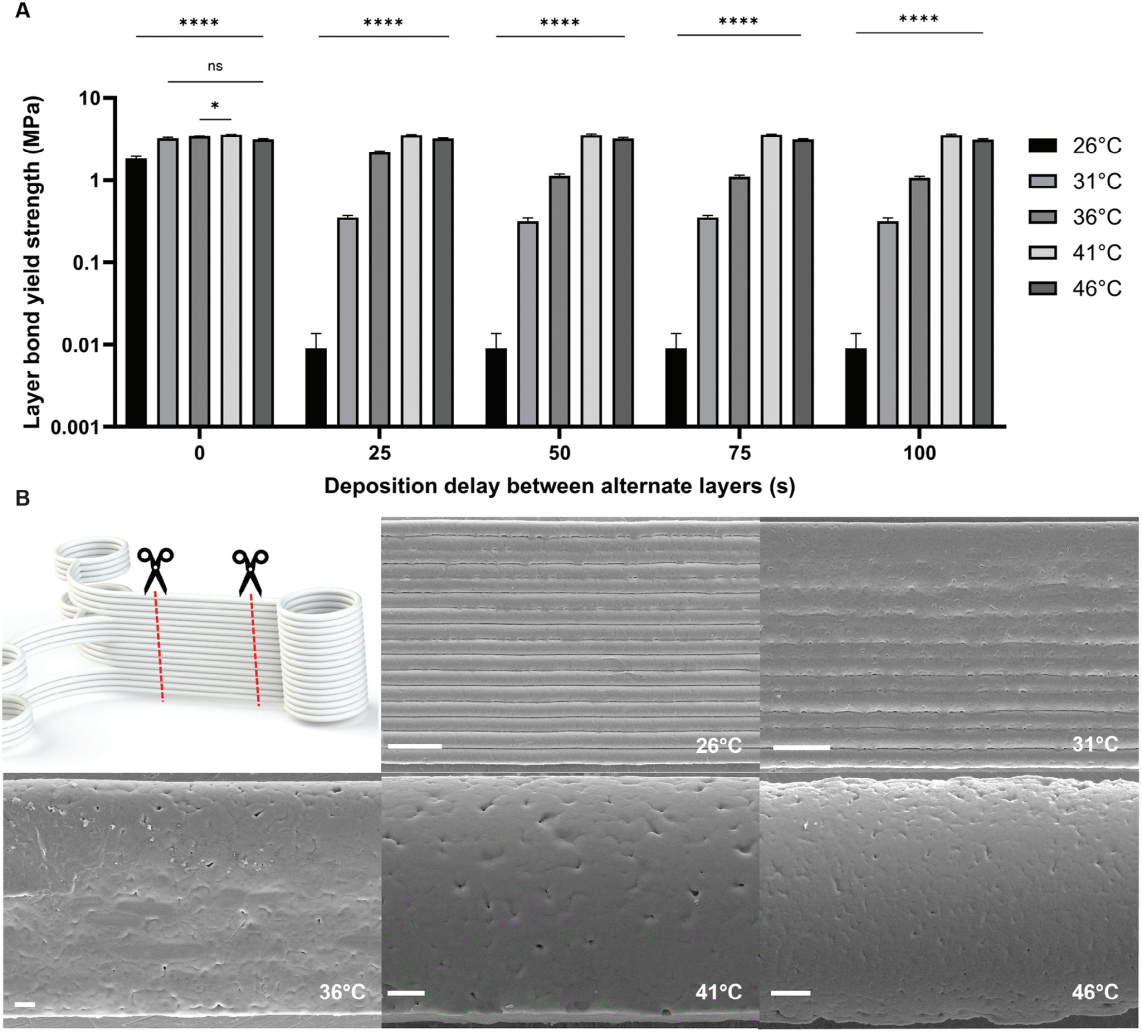
Mechanical test data and scaffold wall imaging. A) Layer bond strength (on a log scale with base 10) for different deposition delays between printing alternate layers for five collector temperatures. All data shows average over *n * = * *5, error bars represent one standard deviation, significant differences were assessed using two‐way ANOVA with Tukey's multiple comparisons test; statistical significance is shown at ^*^ *p* ≤ 0.05, ^****^ *p* ≤ 0.0001, ns = not significant. Unless otherwise specified, all statistical differences are significant (^****^). B) Schematic of sample preparation for SEM investigation, and representative SEM images of scaffolds printed at different collector temperatures (26, 31, 36, 41, and 46 °C). All scale bars = 100 µm.

A significant drop‐off in layer bond strength was found as the deposition delay was increased to 25 s for a collector temperature of 26 °C. This outcome indicates that no polymer bonds were formed between the layers due to the deposited material cooling further with the removal of the insulating print head (**Figure** **4**A).

Increasing the collector temperature to 31 °C without any deposition delay resulted in a 43% increase in layer bond strength. This increase can be attributed to the reduced temperature differential between the nozzle and collector, leading to enhanced fiber bonding. However, with the introduction of a deposition delay, and regardless of the delay time, the layer bond strength decreased and remained at 0.5 MPa for 31 °C. This indicates that despite increases in deposition delay, increasing the collector temperature by 5 °C can improve the formation of polymer bonds, as they are all >40% greater than 26 °C.

At 36 °C, the layer bond strength exhibited a 6% increase compared to 31 °C without deposition delay. Nonetheless, similar to previous collector temperatures, an introduction and increase in the deposition delay resulted in a reduction in layer bond strength. Interestingly, this drop‐off was more gradual, with the layer bond strength decreasing to 1 MPa only after a deposition delay of 50 s or more.

Further raising the collector temperature to 41 °C did not yield a reduction in layer bond strength with increasing deposition delay. This collector's temperature was first to produce a uniform layer bond strength regardless of the delay time. An increase in layer bond strength of 7% compared to 36 °C was observed. The increase in layer bond strength can be primarily attributed to the necking of fibers failing before the layer bonds (Figure S2, Supporting Information).

This increase can be primarily attributed to the increased layer bond strength causing the fibers to neck. This trend was similarly observed at 46 °C, where the fibers again failed before the layer bonds. On the contrary, as the collector temperature was increased from 41 to 46 °C, the layer bond strength decreased by 14%. From the uniaxial tensile test data and images, the decline was attributed to a reduction in tensile strength of the fibers produced a lack of solidification of the previous layer, causing the layers to coalesce and form a solid core with a circular appearance.

To visualize the scaffold wall, each sample was sectioned and laid flat for SEM imaging (Figure 4B). The series of images exhibited a clear visual correlation between increased layer bonding and higher collector temperatures, the scaffolds imaged had no deposition delay. Collector temperatures above 36 °C demonstrated a lack of visible layer lines, yielding a solid wall structure. At 41 and 46 °C, the fibers amalgamated into a large single cylinder, leading to a loss of well‐defined wall shape. Furthermore, this loss of definition was accompanied by a reduction in wall height, declining from 458 µm at 26 °C to 433, 227, 131, and 130 µm at 31, 36, 41, and 46 °C, respectively. Surface features also changed due to increase in collector temperature, producing more spheroid formations above 26 °C.^[^ ^52^ ^]^

It is important to note that the presented quantitative results are largely dependent on the polymer (PCL) and the specific MEW setup. However, our proposed methodology can be extended to accurately determine and optimize layer bond strength using other materials or MEW systems. In addition, we identify collector temperature as an independent parameter to alter layer bond strength without changing any other scaffold properties.

Based on this series of experiments, it is evident that the collector temperature exerts the most significant practical influence on controlling layer bonding, as it can be modified without affecting other processing parameters, including the print path. Furthermore, in addition to collector temperature, the deposition delay findings underscore the importance of considering the scaffold's print path and highlight that scaling up of scaffolds does not guarantee same mechanical properties.

### 2.3 Implications for Larger Scaffolds: Scaffold Architecture Alone is not Responsible for the Overall Mechanical Properties

Building upon the previous findings regarding the sensitivity of layer bonding to deposition delay, we further explored the role of collector temperature in mitigating the resulting reduction in layer bond strength. In particular, we focused on studying the relationship between collector temperature and scaffold strength using a larger scaffold size of 30 × 30 mm. The square scaffolds were designed with 1 × 1 mm pores and consisted of 16 layers. We produced five scaffolds at five collector temperatures.

**Figure 5:**
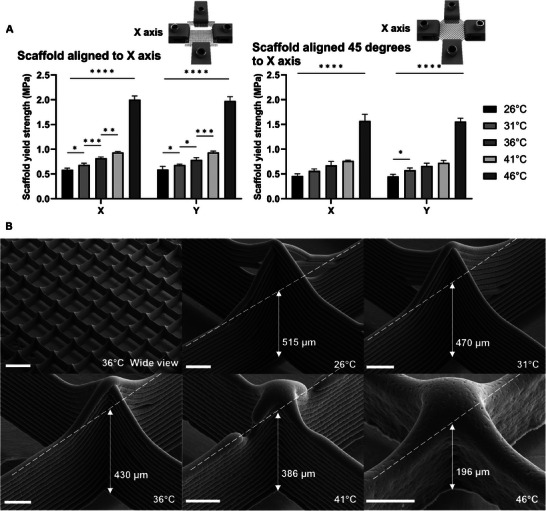
Biaxial testing results showing mounting configuration and SEM images of 30 × 30 mm scaffolds consisting of 16 layers, 1 × 1 mm pores. A) Scaffold yield strength from samples with fibers aligned either parallel or at 45° to mechanical tester axis. Comparing the aligned to the 45° angled scaffolds, for each collector temperature the angled scaffolds produced a lower scaffold strength. All data shows average over *n * = * *5, error bars represent one standard deviation, significant differences were assessed using two‐way ANOVA with Tukey's multiple comparisons test; statistical significance is shown at ^*^ *p* ≤ 0.05, ^**^ *p* ≤ 0.01, ^***^ *p* ≤ 0.001, ^****^ *p* ≤ 0.0001, ns = not significant. B) SEM images of MEW scaffolds printed at collector temperatures of 26, 31, 36, 41, and 46 °C, showing the fiber cross‐over corners. Scale bar: 100 µm, except for 36 °C, which is 200 µm. Wide view image: 1 mm.

Each scaffold was biaxially tested with the direction of the scaffold fibers aligned to the biaxial tester axis (aligned scaffolds) and at 45 degrees (angled scaffolds). By angling the scaffolds, the aim was to exert more force on the nodes. Both scaffold alignments were used to examine if the increased layer bonding increases scaffold strength (**Figure** **5**A). Between both the aligned and angled scaffolds the same increasing trend was observed with a linear increase from 26 to 36 °C, with a rapid increase in strength observed at 46 °C. The variations in scaffold strength observed could be attributed to the reduced cross‐sectional area resulting from increased layer bonding, considering all the tested scaffolds had a similar raw yield force of ≈5N.

For both the aligned and angled scaffolds, a collector temperature of 41 °C produced the highest standard deviation, likely due to the inconsistent defects that appeared at the top of the wall near each node where the top two/three fibers are discontinuous (Figure 5B). These defects can increase the stress concentrations due to the rapid change in cross‐sectional area, causing the scaffold to fail earlier than expected. Thus, these scaffolds presented a lower‐than‐expected layer bond strength.

Upon visual examination of the scaffold nodes, it was observed that collector temperature was able to reduce defects such as fiber separation and scaffold warping. As collector temperature increased, the height of the scaffold nodes decreased due to the increased layer bonding, this caused a reduction in defects at the node where layer separation occurred resulting in gaps in the fiber wall (Figure 5B at 26 and 31 °C). Scaffold warping is caused by the thermal stresses at the edges of the scaffold that are exposed from under the print head during printing.^[^ ^53^ ^]^ The optimum collector temperature for this scaffold print was 36 °C as the defects were almost entirely removed, while the achieved layer bonding improved the scaffold strength by ≈30% and wall height reduction was limited to 85 µm.

The relationship between layer bond strength, scaffold strength, and the impact of different parameters is complex. While there is no direct correlation between the layer bond strength observed during the layer bond mechanical testing and the overall scaffold strength, the increased layer bonding does appear to influence scaffold strength due to the reduction in scaffold height, as evidenced in the layer bond test scaffolds.

The proposed scaffold design for the uniaxial layer bond experiments provides a quick and accurate means to test the effect of parameter combinations on layer bond strength. These experiments identified the collector temperature as the independent parameter that most significantly influences layer bond strength for PCL MEW scaffolds.

It is important to note that mechanical results obtained with the layer bond test scaffolds cannot be directly extrapolated to larger scaffolds, as they will have their own unique printing path and pattern, with different exposure times under the print head or away from it, which will lead to different layer bond strengths. However, this does not diminish the value of the layer bond tests shown previously (Figures 3 and 4) as they serve as a rapid reference for evaluating the effects of new parameter combinations on layer bond strength compared to printing an entire scaffold. Nonetheless, it is essential to perform final printing of the desired scaffolds using the identified processing parameters to ensure the required scaffold strength.

Results demonstrate that collector temperature is an independent parameter suited to tailor layer bonding. By increasing it, the user can increase scaffold strength or even create solid walls within the scaffold, transforming it into a “microwell‐like plate”. Additionally, decreasing the build plate temperature enhances interconnectivity of porous tissue scaffolds. This could be useful in tissue engineering applications where increased porosity is beneficial to facilitate cell migration, vascularization, diffusion or cell‐to‐cell communication across pores.^[^ ^5^, ^23^, ^39^, ^54^ ^]^

We also observed similar effect of collector temperature over layer bonding with scaffolds of small fiber diameters (9 µm) (Figure S3, Supporting Information). Interestingly, the scaffolds with smaller fibers exhibited the same reduction in wall height as the 31 µm fiber diameter scaffolds with increased collector temperature. These results highlight the applicability of this method to scaffolds of any dimensions.

In conclusion, we establish a method to control layer bonding without altering fiber diameter. Moreover, we reveal that scaffold architecture alone is not responsible for the overall mechanical properties, as different layer bond strengths can result in up to 4x increase in the tensile scaffold yield strength.

### 2.4 In Situ Layer Bonding Analysis Through Optical Coherence Tomography

In the context of MEW and the complex interplay of various parameters, there is a growing need for adequate assessment methods to compare properties of scaffolds, particularly in high throughput production environments.^[^ ^19^ ^]^ Currently, such assessment is mainly achieved through destructive techniques like mechanical testing and SEM imaging, which provide accurate readings but irreversibly alter the mechanical properties of the scaffold.

To enable a reliable comparison between newly printed scaffolds and mechanically tested scaffold data, a non‐destructive imaging technique is required to quantify scaffold properties. Through utilizing OCT, it is possible to image the scaffold during or after the printing process and to compare it to a mechanically tested scaffold, thereby assessing an indication of the strength of the layer bonds without destructive testing. This non‐destructive comparison is particularly important to ensure consistent mechanical properties across all scaffolds.

**Figure 6:**
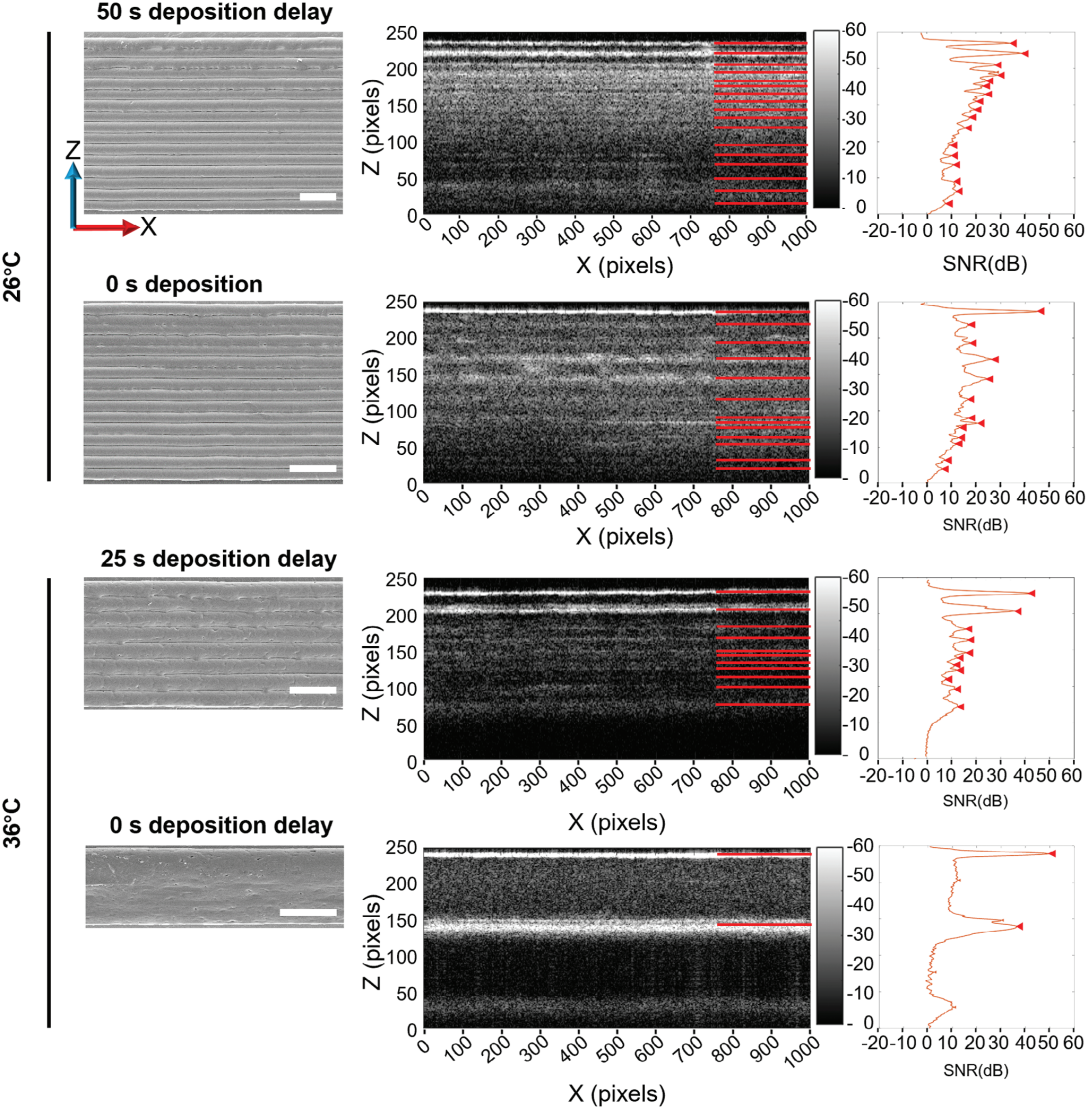
SEM images (left column) with corresponding optical coherent tomography (OCT) scans of the scaffold wall (center); the graphs show the corresponding intensity profile of the signal‐to‐noise ratio (SNR) scan, averaged in the X direction (right column). Red lines on OCT images signify layer lines. The scaffold printed at 26 °C collector temperature and 50 s deposition delay presented the lowest strength layer bond. A medium lower strength layer bond was obtained when printing at 26 °C collector temperature and 0 s deposition delay. A medium higher strength layer bond imaged, printed at 36 °C collector temperature and 25 s deposition delay, and the highest strength layer bond imaged, printed at 36 °C collector temperature and 0 s deposition delay. All scale bars: 100 µm.

The OCT data obtained in this study demonstrated the ability to image 16‐layer scaffolds with a height of up to 0.5 mm and extract the profile of the scaffold wall (**Figure** **6**). Through analysis and noise reduction of the OCT data, the peaks or interfaces between air and fibers, which signify layer bonding, can be detected. It should be noted that the OCT signal attenuates as it passes through the sample, resulting in a decrease in signal amplitude with depth. In each OCT image shown in Figure 6, the red lines indicate the edges of the fibers as observed in SEM images, on the graph the corresponding red triangles show the average line scan signal peak detection.

The first two samples (Figure 6, 26 °C) have the lowest degree of layer bonding, with the resulting average line scan producing 17 and 13 peaks, respectively. The number of peaks detected corresponds to the number of visible layers in the associated SEM images. For the second sample, there are two groups of layers at the top of the SEM image that are completely fused; this fusion is also evident in the OCT image by the lack of peaks detected at the top of the scaffold.

The last two wall profiles (Figure 6, 36 °C) show the samples with the highest degree of layer bonding as shown in the mechanical testing. When printed at 36 °C and 25 s deposition delay, the sample has a reduced OCT peak count in line with the reduced visible layers seen in the corresponding SEM image. The OCT counts 11 layers, which is two more than observed via SEM, which could be caused by noise in the scan or an incorrect peak detection. The signal to noise ration SNR plot from the sample printed at 36 °C without any deposition delay contains 2 large peaks which are consistent with the SEM image showing the top and bottom of the fiber, as all layers are fused together and thus there are no visible layers (horizontal lines) within the OCT images besides the top and bottom ones.

The OCT signal has a limited penetration depth thus visualizing thicker scaffold walls accurately is more challenging due to the signal attenuation. Despite this, our results prove that OCT is a viable method to assess layer bonding qualitatively, and nondestructively in MEW scaffolds thinner than 500 µm. Future work could aim to create a quantitative assessment of layer bonding by correlating the OCT signal to the mechanical test data.

### 2.5 Achieving Heterogeneous Layer Bonding Within a MEW Scaffold

We have so far demonstrated that the collector temperature has a significant role in controlling the overall layer bond strength of an entire scaffold. However, for some applications, variable local scaffold strengths may be desired. Therefore, we next asked whether it was possible to achieve heterogeneous layer bonding within a given MEW scaffold. Unlike 3D FFF printing, MEW is a continuous printing process and therefore the design of the print path must be optimized to allow areas of the scaffold to have different layer bonding without stopping the print jet.

To investigate this, we printed a MEW scaffold with square pores while simultaneously adjusting the collector temperature. For the fibers printed in the X direction, the collector temperature was set at 26 °C, while for those printed in the Y direction, we increased the collector temperature to 41 °C. We repeated this process until we achieved 5 layers of fibers in each direction. Whilst this study was performed by manually varying the collector temperature, this method could be easily automated to improve the efficiency and time associated with this process. It was unclear whether the higher temperature would result in re‐bonding of the already printed layers at cooler collector temperatures, or whether a variable layer bonding could be achieved within the same scaffold.

**Figure 7:**
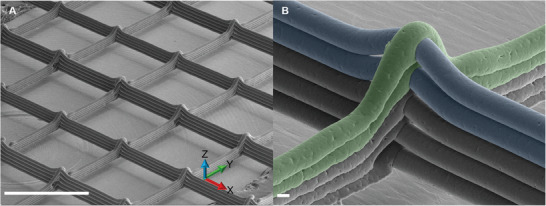
SEM images of a representative PCL MEW scaffold showing variable layer bonding; the scaffold was printed keeping the collector temperature at 26 °C whilst printing fibers in the X direction and increasing the collector temperature to 41 °C for the fibers printed in the Y direction. A) Wide angle view of scaffold showing the print directions. B) High magnification image of a representative node; false color has been used to better identify the fibers printed at different collector temperatures (blue: 26 °C; green: 41 °C). Scale bars: A) 1 mm; B) 20 µm.

Remarkably, SEM revealed a clear difference in layer bonding as shown by the wall height difference in the two printing directions (**Figure** **7**). The wall height in the X direction with cooler collector temperature was 180 µm; and in the Y direction with warmer collector temperature was 130 µm. This is a significant result that demonstrates that it is possible to produce a scaffold with different degrees of layer bonding within a single scaffold, whilst maintaining scaffold shape fidelity and without damaging the already deposited material.

Due to the high heat capacity of the build plate, after changing collector temperature, it was necessary to let it stabilize for 10–15 min, resulting in increased overall print time.

Varying other parameters on the fly such as nozzle temperature could also be used to dynamically alter the layer bonding, ideally using a fully automated MEW setup to avoid inaccurate manual adjustments. However, it is important to keep in mind that they could cause jet instabilities or variable fiber diameters.

## 3 Conclusion

Here we unravel the importance of layer bonding in MEW scaffolds, and present methods for characterizing and controlling it. Results demonstrate that scaffold features such as fiber diameter, the print path of the scaffold design, or printing parameters such as collector temperature have a substantial effect on layer bond strength.

This work presents a robust framework to assess and control layer bonding strength without altering scaffold fiber diameter. This methodology is applicable beyond PCL scaffolds and could be followed with different MEW configurations, although quantitative values of layer bond strength may differ.

Whilst advances in the design of MEW scaffolds have achieved great outcomes in terms of tailoring mechanical properties, our approach provides new possibilities to further accomplish bespoke mechanical properties through controlled layer bonding. Future work could exploit these tools to optimize functional benefits of MEW scaffolds for specific applications using variable layer bonding.

## 4 Experimental Section

### MEW Scaffold Design and Fabrication

All scaffolds were fabricated from medical‐grade poly(ɛ‐caprolactone) (PCL) (Purasorb PC12, Corbion, The Netherlands). Each scaffold was printed at the same temperature and humidity of 23.7 °C ± 1 °C and 38% ± 8%, respectively. All experiments were conducted on a custom‐made MEW printer with the following settings: syringe temperature, 75 °C; nozzle temperature, 85 °C; nozzle length, 1 mm; nozzle diameter, 23G; brass ring diameter, 10 mm; print head diameter, 60 mm; collector size, 200 × 200 mm.

Four collector distances were employed within a small range of 3–4 mm (3, 3.33, 3.66, and 4 mm) to facilitate stable parameter combinations. At each collector distance investigated, applied pressure and applied voltage were varied to obtain two different fiber diameters (25 and 31 µm). Five scaffolds were produced for each combination of fiber diameter, collector distance, and collector speed. To ensure the lag distance remained constant, the MEW printer controller was set to use constant velocity commands that smooths the path profile reducing the number of changes of acceleration, thereby reducing the fluctuations in lag distance.^[^ ^26^ ^]^

For the investigation on the effect of cooling time and collector temperature on layer bonding, five scaffolds per condition were prepared, as per **Table** **2**.

**Table 2:** The experimental printing parameters for each of the different scaffolds tested for the deposition delay study investigating the effect of printing path and print pattern.

Time away from scaffold (s)	0	25	50	75	100
Collector Distance (mm)	3	3	3	3	3
Fiber Diameter (µm)	31 ± 1	31 ± 1	31 ± 1	31 ± 1	31 ± 1
Lag distance (mm)	1.2	1.2	1.2	1.2	1.2
Collector temperature (°C)	26–46	26–46	26–46	26–46	26–46
Collector speed (mm min^−1^)	300	300	300	300	300

For the qualitative assessment of layer bonding through OCT, four different scaffolds were prepared, as per **Table** **3**.

**Table 3:** The experimental printing parameters and yield strength of four different scaffolds used for OCT imaging.

Scaffold name	Low	Medium low	Medium high	High
Collector temperature (°C)	26	26	36	36
Deposition delay (s)	50	0	25	0
Collector distance (mm)	3	3	3	3
Fiber diameter (µm)	31	31	31	31
Lag distance (mm)	1.2	1.2	1.2	1.2
Collector speed (mm/min)	300	300	300	300
Yield strength (MPa)	0.00	1.8	2.2	3.4

### Mechanical Characterization

Mechanical characterization of MEW scaffolds was performed by uniaxial or biaxial testing (Univert, Univert, Cellscale, Canada)^[^ ^49^ ^]^ within the planar XY directions of a printed layer.^[^ ^4^ ^]^ The scaffold design was adapted from (Bakirci, 2021).^[^ ^44^ ^]^ Each scaffold contained three fiber bond regions that were chosen to amplify the force measured due to the small force magnitude found in testing. Every scaffold was clamped to the tester with custom‐made mounting pins (Figure 2). The uniaxial tester was equipped with 1.5 N load cell to be able to detect low magnitudes of layer bonding strengths.

During each test, images were extracted every second to permit visual analysis of the test sample. The force versus displacement graph (Figure S4, Supporting Information) captures the different stages of the mechanical test where i) shows the initial onset of stress once the scaffold becomes taut, ii) shows the yield strength of the sample before destruction of the fiber bonds occurs, iii‐iv) after ii the fiber bonds start to fail, though due to the irregular nature of the bonding along the fiber wall, the data is noisy, until reaching the end of the test region where the bottom loop creates a spike in separation force.

Analysis of the data was performed using a custom Python program that automatically averaged and converted the force‐displacement data to a stress‐strain format, which was used to extract the layer bond yield strength. The cross‐sectional area of the sample wall was calculated using the diameter of a fiber multiplied by the number of layers. The fiber diameters were measured using SEM imaging. The computed cross‐sectional area was experimentally validated by taking a slice of the fiber wall embedded in epoxy and measuring the true cross‐sectional area using optical microscopy. Computed and true values were found to be constant across different conditions due to the consistent fiber diameters obtained (Figure S5, Supporting Information). The true cross‐sectional area was used to determine the stress for each of the samples. Each sample contained three fiber bond regions; thus, the results were normalized to account for them. The bond strength for each parameter combination for all five samples was then analyzed and plotted together. For all mechanical tests, the yield strength refers to the layer bond yield strength for simplicity.

For each collector temperature, ten scaffolds were produced, five samples were mechanically tested with the scaffold walls aligned to the biaxial tester axis and the remaining five will be set at 45° to test the strength of the fiber nodes.

### SEM Imaging

MEW scaffolds were imaged using Zeiss 1555 VPSEM (Zeiss, USA) at acceleration voltage of 15 kV, magnification between 100 and 1000x, at scan speed 2 using frame integration of 210 frames. The low scan speed was used to avoid charging whilst imaging and the high number of frame integration removed noise in the image by combining 210 frames together; each scan took 5 minutes to complete. These settings combined with the largest resolution scan that was supported by the equipment (3072 × 2304) allowed for a high‐resolution scan image.

At each collector temperature investigated, a section of the scaffold was imaged via SEM to show a centered and peripheral node. This was done to assess any potential differences in the layer bonding between these locations, as the center of the scaffold always remained under the print head and that could lead to a greater degree of layer bonding (Figures S6 and S7, Supporting Information). The wall of the scaffold was sectioned and laid flat to permit measurements of the wall height at a node and between two nodes. The wall height between two nodes was used combined with the width of the biaxial testers clamps to determine the scaffold cross‐sectional area (Figure S8, Supporting Information).

### Optical Coherence Tomography Imaging

An ex situ OCT system was set up to image MEW scaffolds. Four samples per group were imaged to assess how well OCT could determine different degrees of bonding. OCT measurements were performed using a fiber‐based spectral‐domain OCT system (TEL220, Thorlabs Inc., USA). The light source is a super luminescent diode with a mean wavelength of 1300 nm and a spectral bandwidth of 170 nm (full width at half maximum (FWHM)). The measured OCT axial resolution in air is 4.8 µm (FWHM). OCT was performed in a dual arm configuration where the OCT reference reflection is provided by a mirror in a separate arm of a Michelson interferometer. The scan lens (LSM03, Thorlabs Inc., USA) has a numerical aperture of 0.063, a measured lateral resolution of 7.2 µm (FWHM), and a working distance of 25.1 mm. Volumetric OCT scans comprised 1000 A‐scans per B‐scan, and 100 B‐scans per C‐scan over a 2 mm × 0.2 mm (XY) lateral region, resulting in a lateral sampling density of 2 µm per voxel.

Each sample was first imaged via OCT, then mechanically tested to determine the yield strength for the layer bonding of that sample and correlate it to the OCT images. Each sample was imaged under the OCT scan head on a metal plate with a gap beneath to remove signal generated by reflection from the surface under the scaffold. Each scaffold was scanned in the same region as shown in (Figure S9, Supporting Information). Once the scaffold was scanned, the OCT data was analyzed using a custom MATLAB script that extracted a signal profile for the wall segment. To permit this and reduce the noise from the scans the scaffold wall was first extracted from the raw OCT and any rotation present in the scan was corrected. Next, the 3D scan data was compressed into a 2D image by averaging the pixels that were across the scaffold wall in the XY plane. Taking a vertical line profile of the scan showed significant noise (Figure S9, Supporting Information). To reduce this noise, the 2D scan data was averaged again along the length of the scaffold wall to produce a single‐line scan that better highlighted the edge of the fibers. A peak detection algorithm was then used to determine the number of peaks that represented the air‐fiber interfaces due to the scattering of the light from the OCT beam.

### Statistical Analysis

All data unless stated otherwise is for *n* = 5 samples shown as the mean ± standard deviation. For mechanical testing both uniaxial and biaxial data, statistical analysis was performed using a two‐way ANOVA with Tukey's multiple comparisons test. Alpha was 0.05 for all tests. All statistical analysis was conducted using GraphPad Prism software (GraphPad, San Diego, USA).

## Conflict of Interest

The authors declare no conflict of interest.

## Supporting information



Supporting Information

Supplemental Video1

## Data Availability

The data that support the findings of this study are available from the corresponding author upon reasonable request.
