# Supplementary material for: Understanding the Significance of Layer Bonding in Melt Electrowriting
Source: Adv Sci (Weinh). 2024 Oct 24;11(47):2407514. doi: 10.1002/advs.202407514 (PMC11653759; doi:10.1002/advs.202407514)
Supplement: Supplementary file 1 — Supporting Information [file ADVS-11-2407514-s001.docx]

# Supporting Information

#
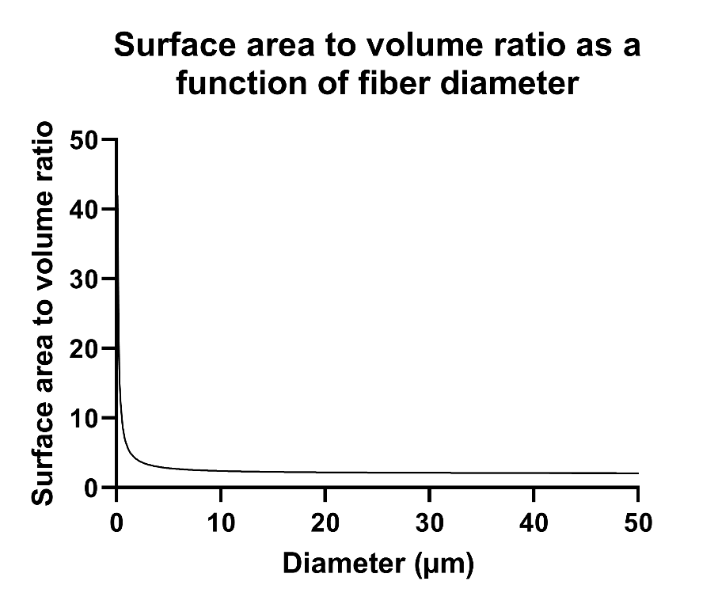


# Figure S1. Surface area to volume ratio as a function of fiber diameter


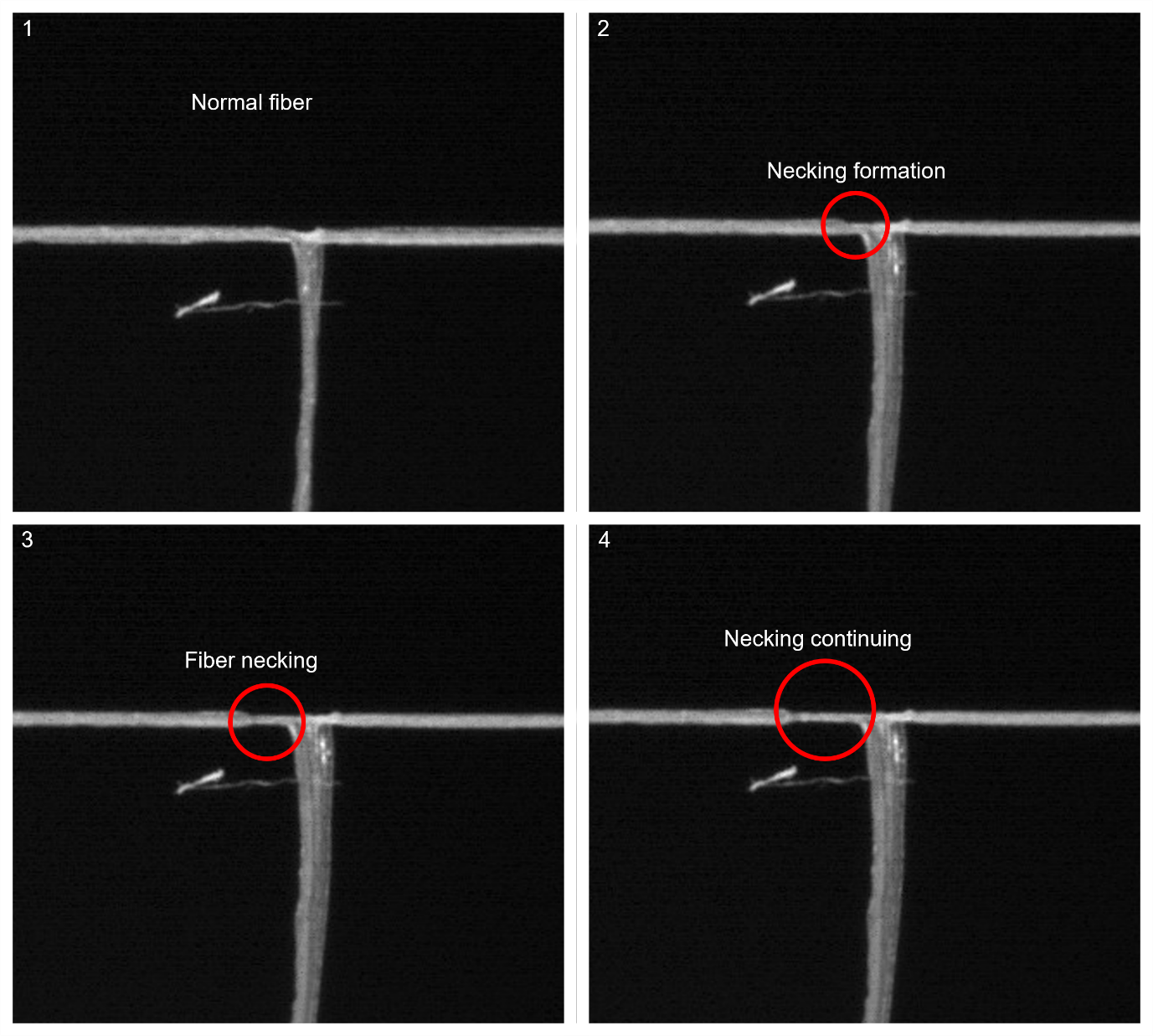


# Figure S2. Plastic failure and formation of necking in PCL fibers of the scaffolds used for layer bonding studies during uniaxial tensile mechanical testing. A) Fiber under uniaxial tensile tension. B) Necking formation initiates when stress exceeds the yield strength of PCL. C) Fiber necking continues to propagate. D) PCL fiber does not completely break but necking results in a significant reduction of cross-sectional area.

#
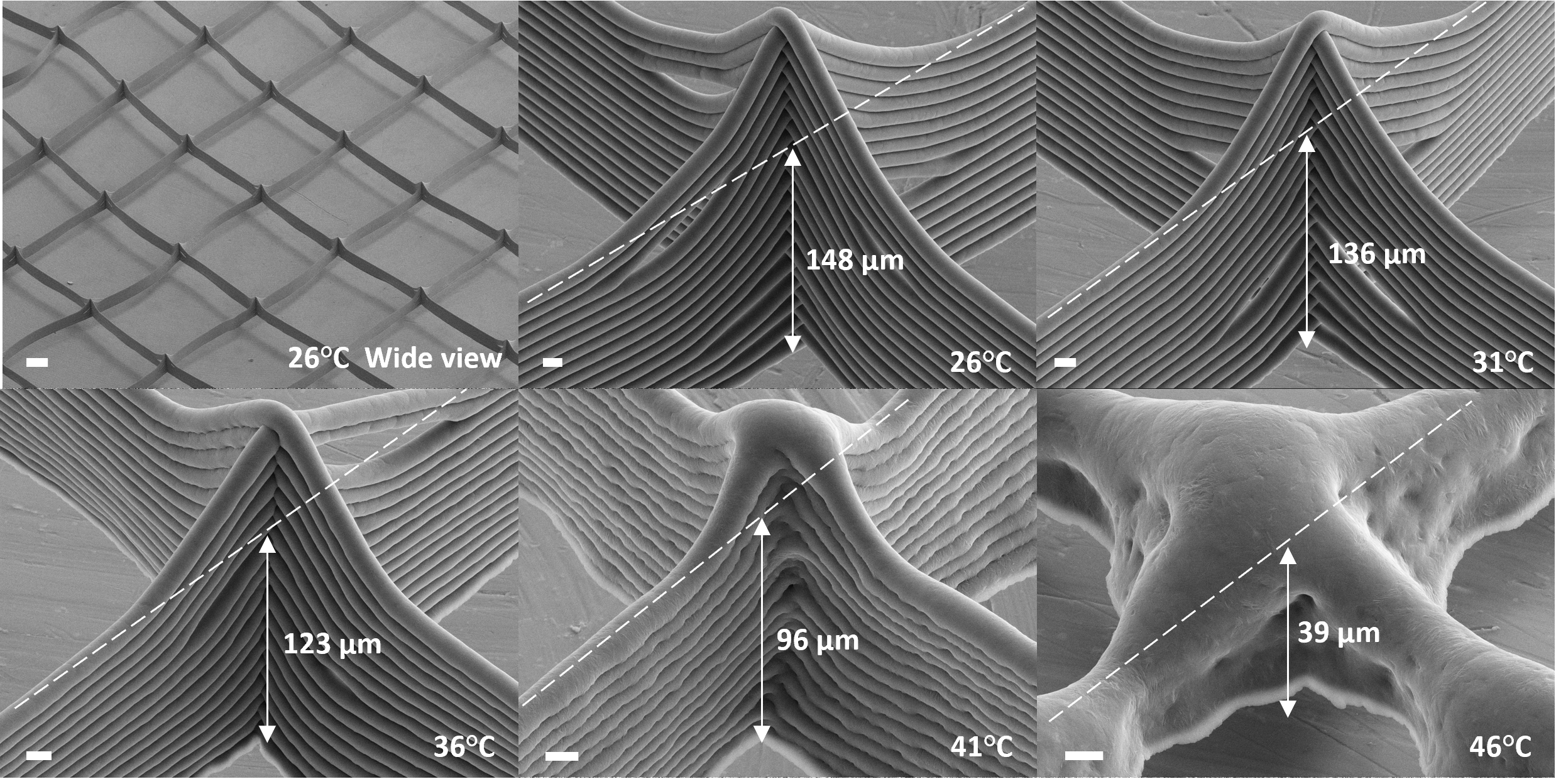


**Figure S3.** SEM images of MEW scaffold with small fiber diameter (9 µm ± 0.5 µm) printed at collector temperatures of 26, 31, 36, 41, and 46°C, showing the fiber cross-over corners. Scale bar: 10 µm, except for wide view image: 100 µm.


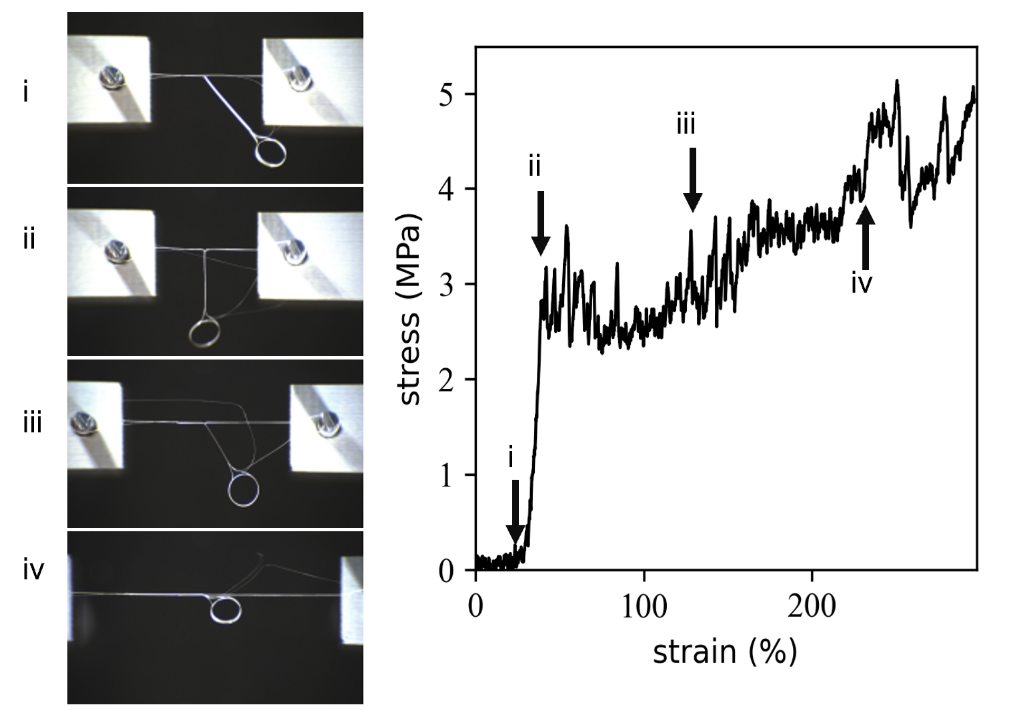


**Figure S4.** Sequence of images taken at different timepoints during uniaxial tensile tests and corresponding stress vs strain curve indicating those timepoints (i-iv).


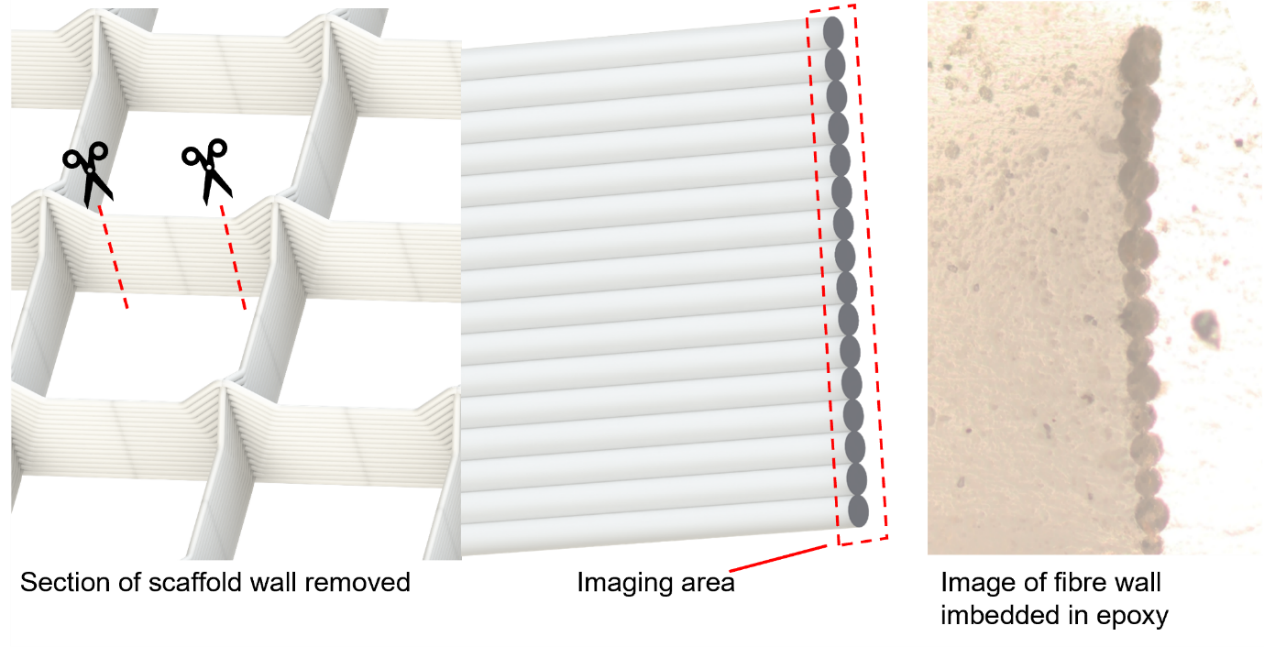


**Figure S5.** Diagram explaining the method used to measure experimentally the cross-sectional area of the scaffold wall. A section of the scaffold wall is first removed and then embedded in epoxy, which is sectioned perpendicular to the direction of the fibers and optically imaged. The cross-sectional area is extracted from the image using a custom python script.

#
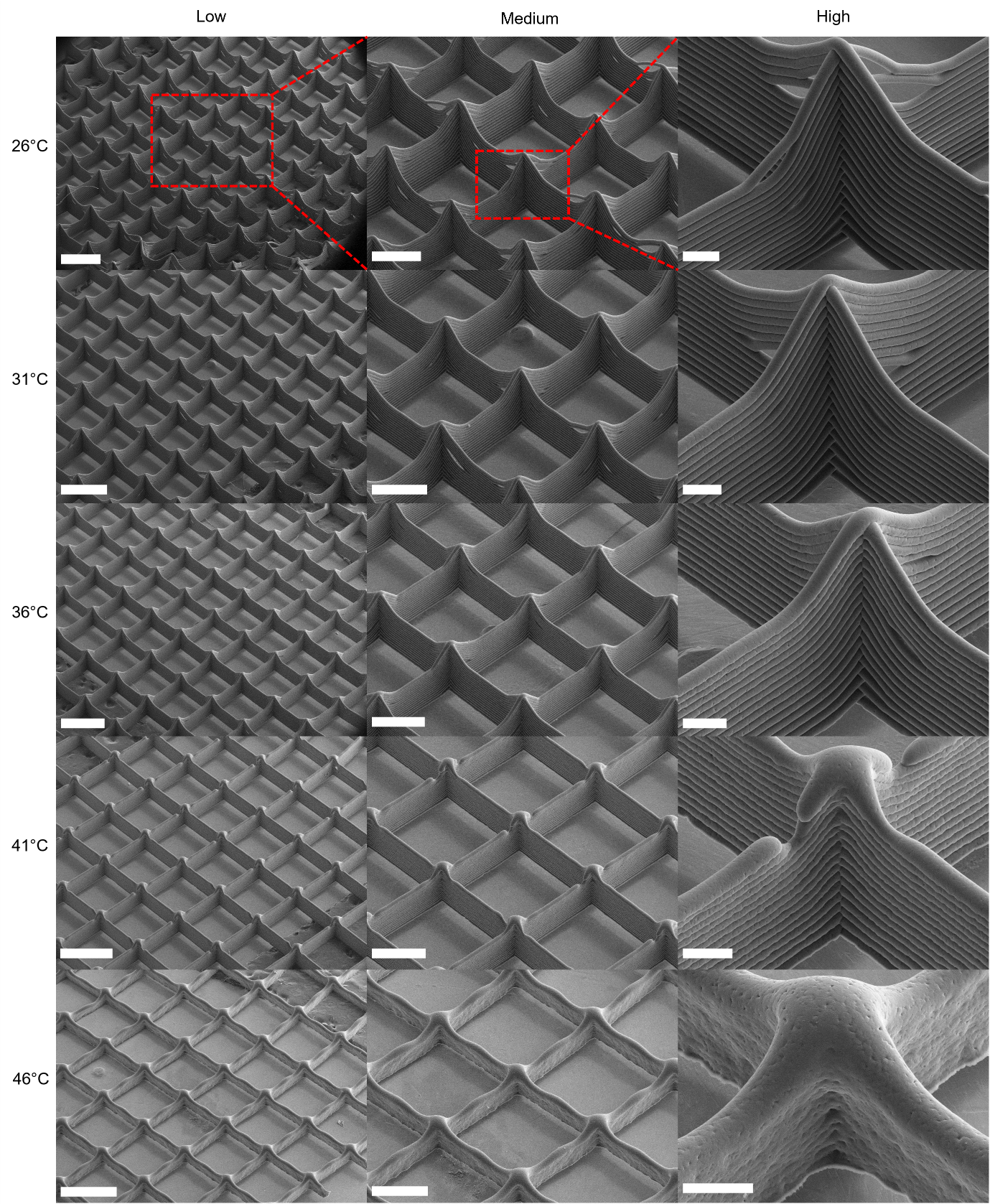


# Figure S6. Scanning electron microscopy images taken from the center of representative 30 × 30 mm scaffolds, left to right is low (50x), medium (120x), and high magnification (400x), printed using collector temperatures of 26, 31, 36, 41 and 46°C. Scale bars for low magnification: 1 mm; medium magnification: 500 µm; high magnification: 100 µm.


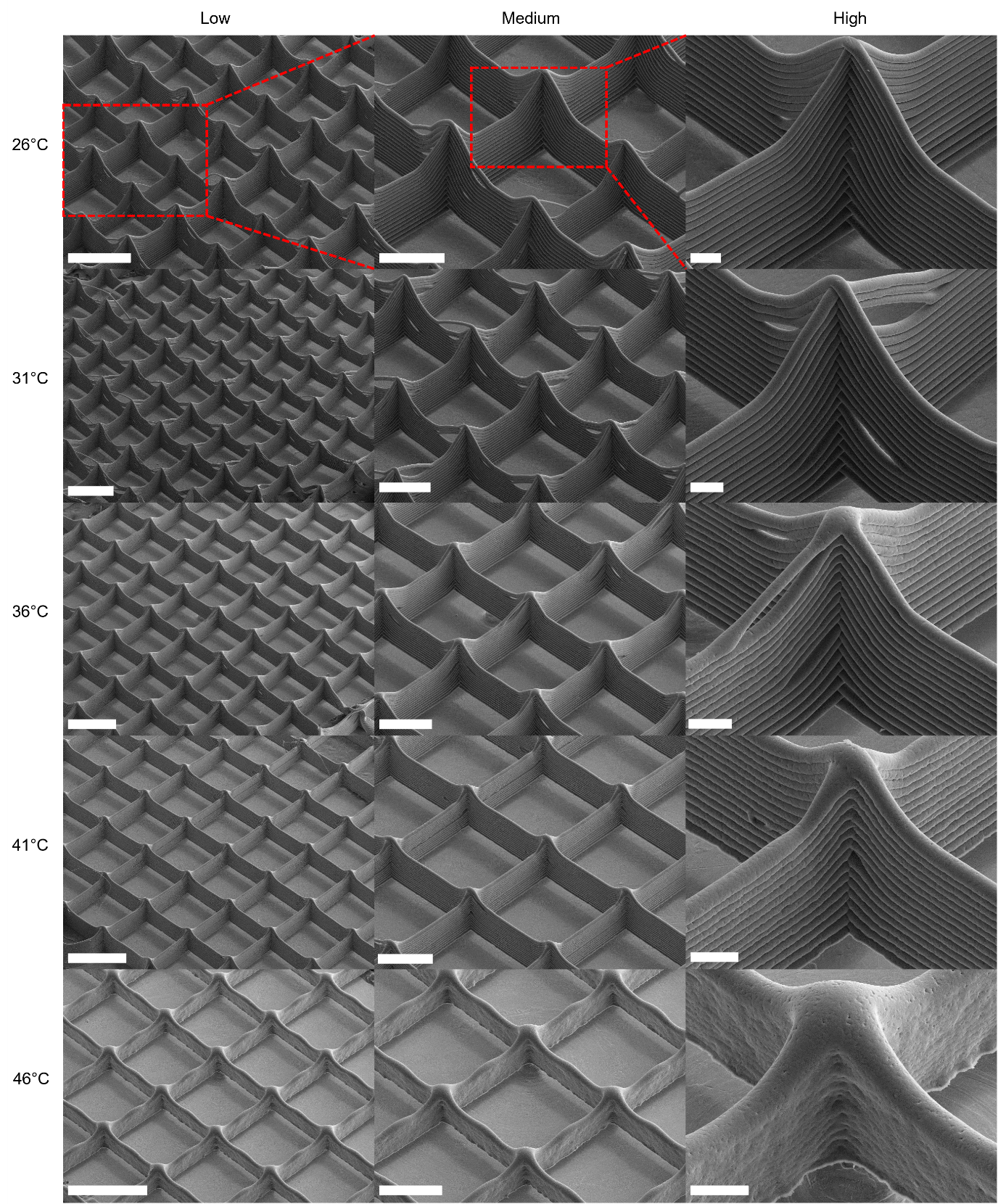


**Figure S7.** Scanning electron microscopy images taken from the edge of representative 30 × 30 mm scaffolds, left to right is low (50x), medium (120x), and high magnification (400x), printed using collector temperatures of 26, 31, 36, 41 and 46°C. Scale bars for low magnification: 1 mm; medium magnification: 500 µm; high magnification: 100 µm.

#
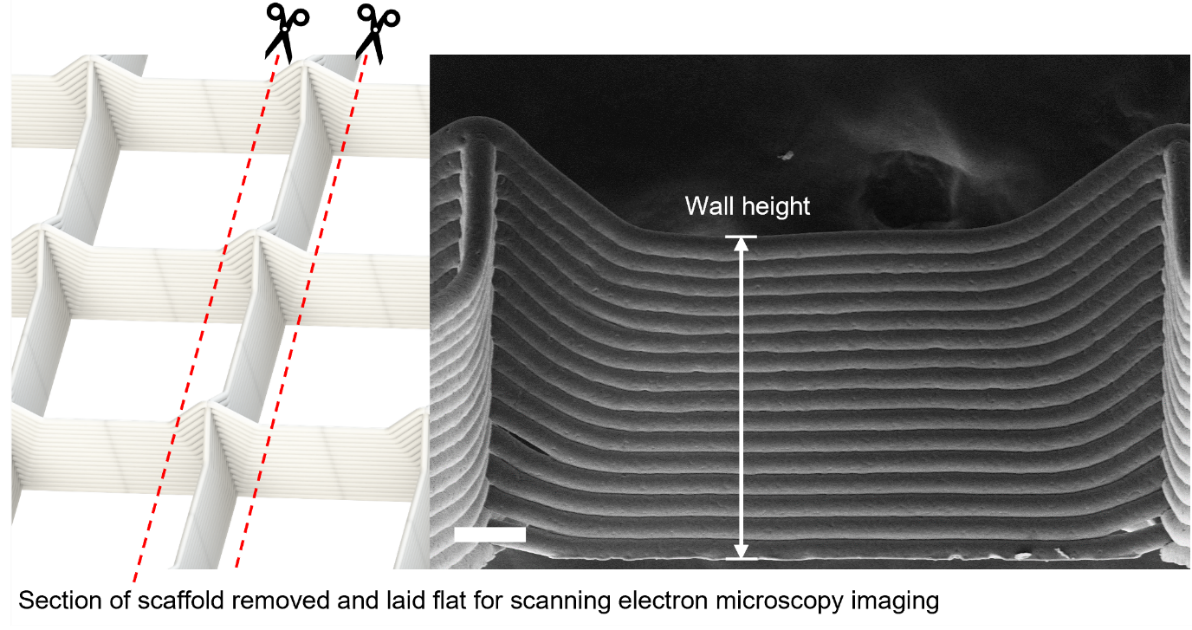


# Figure S8. Scaffold sectioning method used to determine the wall height via scanning electron microscopy in the 30 × 30 mm scaffolds. Scale bar: 100 µm.


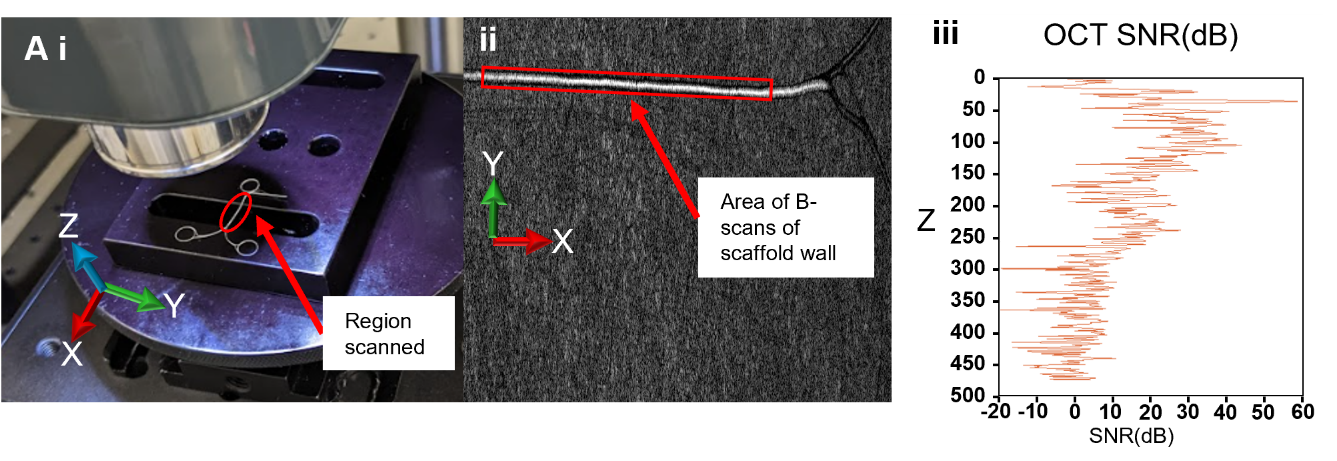


**Figure S9.** i) View of optical coherence tomography scan head and scaffold location, with scaffold region to be scanned circled. ii) Top-down view of the scaffold wall showing the area at which OCT B-scans are taken. iii) Representative OCT A-scan showing the signal-to-noise ratio (SNR) as a function of the wall thickness (i.e., penetration depth, Z, in pixels).

**Video S1.** Rendered video of the print path programmed to manufacture the scaffolds used for the layer bonding tensile testing.
